# Impact of late radiation effects on cancer survivor children: an integrative review

**DOI:** 10.1590/S1679-45082015RW3102

**Published:** 2016

**Authors:** Cibeli Fernandes Coura, Patrícia Cláudia Modesto

**Affiliations:** 1 Hospital Israelita Albert Einstein, São Paulo, SP, Brazil.

**Keywords:** Neoplasms/radiotherapy, Neoplasms/radiation effects, Child

## Abstract

We aimed to identify the late effects of radiation exposure in pediatric cancer survivors. An integrated literature review was performed in the databases MEDLINE and LILACS and SciELO. Included were articles in Portuguese and English, published over the past 10 years, using the following keywords: “*neoplasias*/neoplasms” AND “*radioterapia*/radiotherapy” AND “*radiação*/radiation”. After analysis, 14 articles - published in nine well-known journals - met the inclusion criteria. The publications were divided into two categories: “Late endocrine effects” and “Late non-endocrine effects”. Considering the increased survival rates in children who had cancer, the impact of late effects of exposure to radiation during radiological examinations for diagnosis and treatment was analyzed. Childhood cancer survivors were exposed to several late effects and should be early and regularly followed up, even when exposed to low radiation doses.

## INTRODUCTION

The incidence of cancer worldwide has increased considerably, thus becoming a global public health issue. According to the World Health Organization, it is estimated that 21.4 million new cancer cases will be diagnosed by 2030. In Brazil, approximately 576 thousand new cancer cases were expected in 2014 and 2015.^1^

Pediatric tumors account for 1-3% of all cancer cases in the world. Therefore, 11,840 new cancer cases were expected in Brazil in 2014 and 2015, representing one of the leading causes of death among children and adolescents.^1^

Cancer can be treated with surgery, chemotherapy and radiation therapy (RT). Technological advances in treatment modalities led to a significant impact on the overall survival of these patients. Currently, 80% of the patients can be cured if diagnosed early.^2^

RT is one of the most commonly used therapeutic modalities to reduce or prevent tumor growth. Exposure of normal tissues, however, may result in cancer. Ionizing radiation damages human tissues, producing irreversible chemical and biological changes, and resulting in cell death.^3^

Concomitantly to this treatment modality, short and long-term late effects of radiation exposure have been observed, resulting from the treatment and radiation-based diagnostic tests in children and adolescents. The side effects of this exposure can cause endocrine and non-endocrine disorders, modifying the nutritional status of individuals, and making them more susceptible to complications.^4^

Thus, early and regular follow-up is necessary to enable the diagnosis of these late side effects, thereby providing improved quality of life, considering the high rate of survival of these patients.

## OBJECTIVE

Identify the late effects of radiation exposure in children surviving cancer.

## METHODS

We conducted a research study based on an integrative review including theoretical and empirical literature, as well as studies with different methodologies. To prepare this study, the following steps were followed: establishment of the hypothesis and objective of the integrative review; definition of inclusion and exclusion criteria for sample selection; definition of the information to be extracted from articles; analysis of results; discussion and interpretation of results; and presentation of the review.^5^

The guiding question chosen was: what are the late effects of radiation exposure in children surviving cancer?

We conducted searches in electronic journals found in databases Medical Literarure Online (MEDLINE) and Latin Americam and Caribbean Center on Health Sciences (LILACS), and electronic support Scientific Eletronic Library Online (SciELO).

To search for articles, we used the following descriptors: “*neoplasias*/neoplasms” AND “*radioterapia*/radiotherapy” AND “*radiação*/radiation”. These descriptors can be found in the Health Sciences Descriptors by BIREME.

The inclusion criteria for sample selection were: articles available online in full text, in Portuguese and English, published over a 10-year period (2003-2013), covering the late effects of radiation exposure in children surviving cancer. We excluded articles in duplicate, and those that did not meet the study objective.

After crossing the descriptors in the online databases, we found 452 articles, which were initially evaluated based on the title and abstract; when compatible with the guiding question and inclusion criteria, they were read in full. After critical analysis, the final sample was composed of 14 articles.

For data collection, the authors of this research study prepared a structured script consisting of the following information: identification of the articles, type of publication, language, author, year of publication, title, population, objective, results and conclusions.

The data were analyzed according to the content and, after assessment of the material, articles were grouped and identified under thematic categories.

Results presentation and data discussion were made in the form of descriptions and tables, enabling readers to apply the results of the integrative review proposed.

## RESULTS

Of the 452 articles initially evaluated by title and abstract, 61 were read in full because they were compatible with the guiding question and the study objective. After critical analysis, 44 articles were excluded for not meeting the inclusion criteria and three for duplicate indexing. Therefore, this review encompassed 14 articles.

Among these articles, 12 were found in the MEDLINE database (86%), one in LILACS (7%) and one in SciELO (7%). As for the year of publication, two were from 2012, three from 2011, four from 2010, two from 2009, one from 2008, one from 2006 and one from 2005.

English was the predominant language in the publications, with 12 articles (86%).

When looking into the type of study, six were retrospective reviews (two longitudinal studies and four cohort studies, with one observational and three longitudinal studies), two articles were literature reviews, and two were systematic reviews.

The articles selected were published in seven reputable journals: Cancer (three articles); *Arquivo Brasileiro de Endocrinologia & Metabologia* (two articles), Radiology and Radiation Research (two articles); Archives of Internal Medicine (one article); Pediatr Blood Cancer (two articles) and The Lancet (one article).

Table 1 shows the categories and the number of publications identified in this study.

**Table 1:** Description of categories

Category	Number of publications
Endocrine late effects	7
Non-endocrine late effects	10
Total	17

The publications were divided into two thematic categories: “Endocrine late effects” and “Non-endocrine late effects”. Of the 14 articles selected, seven fell under category “Endocrine late effects” and 10 under “Non-endocrine late effects” (three articles appeared in both categories).

Chart 1 shows the articles by author, year of publication, title, type of publication and results.

**Chart 1:** Description of the articles

Author	Title	Type of study	Results
Bonato et al.^(3)^	[Thyroid disorders associated with external radiation in children and adolescents]	Literature review	Radiation exposure in children resulting from radiation-based diagnostic exams caused hypothyroidism and thyroid cancer
Couto-Silva et al.^(4)^	[Endocrine sequelae after RT in childhood and adolescent cancer]	Literature review	High doses of radiation caused growth hormone deficiency, obesity, hypothyroidism, gonadal dysfunction and marked height deficit
Armstrong et al.^(6)^	Long-term effects of radiation exposure among adult survivors of childhood cancer: results from the childhood cancer survivor study	Longitudinal retrospective review	RT was associated with increased risk of late mortality, neoplasms, obesity, lung dysfunction, heart failure and hypothyroidism
Cheuk et al.^(7)^	Prognostic factors and long-term outcomes of childhood nasopharyngeal carcinoma	Retrospective review	Patients with nasopharyngeal carcinoma who received RT >30 Gy for the primary tumor developed subsequent neoplasms and morbidities
Bhatti et al.^(8)^	Risk of second primary thyroid cancer after radiotherapy for a childhood cancer in a large cohort study: an update from the childhood cancer survivor study	Longitudinal retrospective cohort	The risk of a second primary thyroid cancer in survivors of childhood cancer increased with radiation doses up to 20 Gy, with with a 14-times-higher peak relative risk
Meacham et al.^(11)^	*Diabetes mellitus* in long-term survivors of childhood cancer. Increased risk associated with radiation therapy: a report for the childhood cancer survivor study	Longitudinal retrospective cohort	Three types of RT (TBI, abdominal and cranial) were compared in association with *diabetes mellitus*. Patients treated with TBI were seven times more likely to develop *diabetes mellitus*
Wallace^(12)^	Oncofertility and preservation of reproductive capacity in children and young adults.	Retrospective review	Children exposed to total body irradiation (TBI), abdominal radiation or pelvic radiation showed impaired fertility in both genders
Grewal et al.^(13)^	Auditory late effects of childhood cancer therapy: a report from the children’s Oncology Group	Systematic review	High-dose cranial RT resulted in ototoxicity, impairing quality of life in children
Motosue et al.^(15)^	Pulmonary function after whole lung irradiation in pediatric patients with solid malignancies	Longitudinal retrospective review	Lung irradiation caused pulmonary complications, and patients treated with radiation impulses had increased morbidity and metastasis
Jones et al.^(17)^	Renal late effects in patients treated for cancer in childhood: a report from the Children’s Oncology Group	Systematic review	Subjects treated with radiation for childhood cancer were at risk for kidney failure. Doses >20 Gy resulted in kidney disease
Ritchey et al.^(18)^	Late effects on the urinary bladder in patients treated for cancer in childhood: a report from the Children’s Oncology Group	Retrospective literature review	High-dose radiation in the pelvic region increased the risk of bladder function abnormalities such as hemorrhagic cystitis, fibrosis and neurogenic bladder
Taylor et al.^(19)^	Population-based risks of CNS tumors in survivors of childhood cancer: the British Childhood Cancer Survivor Study	Longitudinal retrospective cohort	Increased risk of a second primary tumor in the central nervous system was demonstrated after exposure to radiation in meningeal tissues
Kleinerman^(20)^	Cancer risks following diagnostic and therapeutic radiation exposure in children	Retrospective review	Exposure to multiple imaging exams increased the risk of cancer in children due to the radiation inherent to the procedure
Pearce et al.^(21)^	Radiation exposure from CT scans in childhood and subsequent risk of leukaemia and brain tumours: a retrospective cohort study	Observational retrospective cohort	The use of CT scan in children accumulated radiation doses of 50 Gy, which could triple the risk of leukemia, and doses of 60 Gy which could triple the risk of brain cancer

RT: radiation therapy; TBI: total body irradiation; CT scan: computed tomography scan ; CNS: central nervous system.

## DISCUSSION

### Endocrine late effects

Endocrine sequelae were described after radiation exposure in patients subjected to cancer therapy, including hypothyroidism, growth hormone (GH) deficiency, obesity, diabetes and gonadal disorder.^3,4,6-12^

Thyroid conditions are the most frequent endocrine complications. In children irradiated in the area, hypothyroidism was found in 47% of patients, 27 years after treatment, and half of these cases occurred up to 5 years after RT.^3,4^ Hypothyroidism was also identified in 34% of 1,791 survivors of Hodgkin’s lymphoma with a relative risk (RR) of 17.1.^6^ Other authors showed that 30% of patients receiving RT doses of 35 to 45 Gy and 50% of those receiving doses >45 Gy would develop hypothyroidism 20 years after treatment, and the estimated risk of nodules was 27 times higher.^4,6^ The cumulative incidence in a 15-year estimated of primary hypothyroidism in patients receiving RT doses >60 Gy was 77.2%; for doses of 50 to 60 Gy, 47.4%; and for doses <50 Gy, 9.5%.^7^ In the short term, 39% of children treated with irradiation of different body areas developed hypothyroidism in average 3.5±1.9 years post RT.^3^

Adenomatous nodules were observed in 30-90% of patients irradiated in the head and neck, with papillary carcinoma as the most common in 78% of cases of second thyroid cancer.^3^ In a cohort of 17,980 survivors of childhood cancer, the RR was 4.6, with a 95% confidence interval (95%CI) for the appearance of a second thyroid cancer, when compared to survivors not treated with RT.^3^ Of 119 children treated for childhood cancer, all had a second primary thyroid cancer and the risk increased with radiation doses between 20 and 25 Gy, with a 14.6 times higher peak RR with 95%CI of 6.8-31.5.^8^

Cranial RT also led to GH deficiency. Among the patients receiving doses of 24 Gy, 56% had GH deficiency, and among those receiving doses >45 Gy, 100% had GH deficiency.^4^ The decrease in GH levels was observed right after 18 months in those receiving doses of 31 to 42 Gy, and 60% had GH deficiency after two years. Once the GH deficiency was established, a marked delay in height growth was observed at doses >45 Gy; at doses <24 Gy, the delay was inconsistent. Height deficit with trunk shortening was observed in patients subjected to spinal irradiation at doses >20 Gy.^4^ The same was confirmed in another study, in which 100% of children receiving doses >30 Gy had GH deficiency, and isolated GH deficiency can occur at doses as low as 18 Gy. Also, these children had higher risk of stunting, which confirmed the presence of growth delay in these patients.^9^

Obesity was a risk predictor of diabetes, dyslipidemia, hypertension and cardiovascular disease, and was associated with cranial radiation at doses between 20 and 24 Gy.^6^ Patients who received cranial RT at 20 Gy (mean dose of 24 Gy) had increased risk of overweight and obesity, and those who received doses >45 Gy showed increased body composition, thus confirming that the weight gain was dose-dependent.^10^

Survivors subjected to abdominal irradiation were 2.7 times more likely to develop *diabetes mellitus* when compared to non-treated patients, and those subjected to total body irradiation (TBI) were 7.2 times more likely to have *diabetes mellitus.* ^11^

Gonadal dysfunction after cranial, abdominal and pelvic RT or TBI impaired fertility in both genders.^4,12^ Precocious puberty occurred after doses between 18 and 24 Gy. Doses <4 Gy did not lead to permanent ovarian dysfunction; doses >20 Gy and TBI led to ovarian failure in most children and adolescents.^4^ Among the patients who, during childhood, received doses of 10 to 15.75 Gy after head computed tomography scanning (CT) scanning, 90% had ovarian failure; of those subjected to abdominal irradiation between 20 and 30 Gy, 97% had ovarian failure with increased risk of premature menopause. Ovarian failure and early menopause were observed in the Childhood Cancer Survivor Study (CCSS), a cohort of 14,000 survivors of childhood cancer treated with pelvic irradiation. Exposure during pregnancy increases the risk of miscarriage, premature birth and low birth weight.^12^

In men, low radiation doses between 0.1 and 1.2 Gy may adversely affect spermatogenesis; and doses >4 Gy caused permanent azoospermia. Leydig cells were more resistant than germ cells, and damage to the former was only observed at doses >20 Gy in pre-pubescent boys and at 30 Gy in post-pubescent boys.^12^

### Non-endocrine late effects

RT played an essential role in the treatment of different childhood neoplasms, increasing patient survival, but has also been linked to several deleterious effects such as hearing loss and different organ dysfunctions (*eg.* lung, heart, kidney and bladder), as well as development of second neoplasms.^6,7,13-19^

Cranial RT at doses >32 Gy has been found to cause ototoxicity in pediatric patients, with progressive hearing loss manifesting months or years after exposure. Ototoxicity could also be seen in 34% of patients, and grades 3 and 4 were reported in 9% of patients who received RT prior to chemotherapy.^13^ The CCSS study confirmed the hearing loss after doses >30 Gy (RR.: 7.5; 95%CI: 3.6-15.5). With doses >50 Gy, the risk increased (RR: 10.7; 95%CI: 5.2-22.2), corroborating the ototoxicity.^14^

Chest irradiation and TBI caused pulmonary toxicity with RR of 4.3 for pulmonary fibrosis, 2.2 for recurrent pneumonia and 2.0 for chronic cough.^6^ In 48 survivors of pediatric solid tumors with a mean follow-up of 9.7 years treated with whole lung irradiation with a mean dose of 12 Gy, there was significant decrease in lung capacity. Also, 17 of these patients received lung irradiation by focal impulse with a mean dose of 18 Gy, which increased the frequency of lung morbidity as well as metastatic disease.^15^

Radiation exposure of the heart at 15 Gy led to two- to six-times greater risk of congestive heart failure and myocardial infarction, when compared to non-exposed survivors. Those receiving chest or spine irradiation or TBI had three-fold higher risk of cardiac death (RR: 3.3; 95%CI: 2.0-5.5).^6^ It was also possible to confirm that heart RT at 20 Gy led to 4.40-fold higher risk of heart disease (95%CI: 1.0 17.5) when compared to non-exposed patients, and the RR for heart failure of those receiving a mean dose of 5 to 20 Gy was 2.52, with a 95%CI of 0.96-6.6. At doses >20 Gy, the RR was 5.65 with a 95%CI of 1.45-22.0, thus confirming the high risk for morbidity and mortality.^16^

Renal function was assessed in patients treated for childhood cancer, and it was found that in patients subjected to kidney irradiation, nephropathy or nephritis occurred after 3 to 12 months, manifesting with hypertension, proteinuria, anemia and kidney failure. Doses <18 Gy in the kidney rarely caused serious injury; however, doses >20 Gy resulted in significant nephropathy.^17^

Radiation-induced hemorrhagic cystitis may be acute or chronic, at doses >30 Gy in the whole bladder and doses >60 Gy in a portion of the bladder. Among 11 children aged 6 years or older treated for pelvic rhabdomyosarcoma, 7 were irradiated and presented with enuresis and abnormal bladder capacity. In another 40 patients with the same disease, 9 of the 19 irradiated patients had bladder problems; those receiving doses of 40 Gy had higher incidence of urinary symptoms, and everyone receiving doses >50 Gy had bladder complications. RT >50 Gy in the lumbar spine or sacrum resulted in damage to peripheral nerves leading to neurogenic bladder.^18^

Subsequent neoplasms were observed in 5 of 59 patients with nasopharyngeal carcinoma subjected to RT at 30 to 70 Gy.^7^ In the CCSS cohort, 298 survivors developed malignancies subsequently to radiation exposure with a cumulative incidence of 3.2% over 20 years after the primary diagnosis of childhood cancer. A study of 6,068 female survivors found that 95 had secondary breast cancer after chest irradiation with a standardized incidence ratio (SIR) of 24.7 and 95%CI: 19.3-31.0; when compared with those not receiving RT, the SIR was 4.8 (95%CI: 2.9-7.4). Also, chest irradiation at 40 Gy was observed to increase the risk of breast cancer by 11 times.^6^ The literature also identified 247 second primary neoplasms of the central nervous system, including 137 meningiomas, 73 gliomas and 37 other types of cancer. The SIR was 14.3 (95%CI: 10.9-18.7) after RT; when compared to those not receiving RT, the SIR was 6.1 (95%CI: 3.1-11.0). In patients irradiated at 40 Gy, the adjusted RR for meningioma was 479 times higher than for non-exposed patients.^19^

### Late effects of diagnostic exams

An association between diagnostic exams and cancer has been reported.^3,20,21^ Although the radiation doses of one single procedure may be low, pediatric patients are subjected to multiple exams, resulting in relatively high cumulative doses. In respect to radiation exposure of the chest due to multiple fluoroscopies used to monitor tuberculosis treatment in adolescent girls and young women, with a mean fractionated dose of 0.79-2.1 Gy, breast cancer appeared 15 years after exposure and remained high until 50 years later.^20^ Dosimetry tests in children subjected to neck CT scans showed that the thyroid can receive doses of 15.2 to 52 Gy, which could increase malignancy cases by more than 390 in every million people exposed.^3^ In a cohort of children and young adults non-diagnosed with cancer and subjected to CT scans, 74 of 178,604 patients were diagnosed with leukemia and 135 of 176,587 patients were diagnosed with brain tumor. The RR of leukemia in patients receiving a mean dose of 51.13 mGy was 3.18, compared to those receiving <5 mGy. But the RR of brain tumor in patients receiving a mean dose of 60.42 mGy was 2.82, and 3.32 in those receiving a mean dose of 104.16 mGy, compared to those receiving <5 mGy. CT scans in children <15 years of age with cumulative radiation doses of 50 mGy can triple the risk of leukemia, and cumulative doses of 60 mGy can triple the risk of brain tumor.^21^

The search methodology used in this study has limitations related with the choice of descriptors and the date range, which resulted in a very broad and poorly sensitive search. Another parameter was related to the difficulty obtaining more samples due to lack of access to paid articles. All these factors were limiting, and a more significant and representative sample would certainly increase the validity of the study.

## CONCLUSION

The authors show that, despite the advances in cancer therapies, alarming short and long-term side effects associated with radiation exposure have been observed in association with treatments and diagnostic tests. Survivors were exposed to several late effects, such as hypothyroidism, GH deficiency, obesity, *diabetes mellitus,* infertility, hearing loss, different organ dysfunctions, such as lung, heart, kidney and bladder, in addition to development of a second neoplasm. Thus, we emphasize the importance of early and regular follow-up of patients exposed to radiation during childhood, even at low doses, in order to enable diagnosis and treatment of these side effects, leading to better quality of life.
