# Supplementary material for: Impact of late radiation effects on cancer survivor children: an integrative review
Source: Einstein (Sao Paulo). 2016 Jan-Mar;14(1):71–6. doi: 10.1590/S1679-45082015RW3102 (PMC4872922; doi:10.1590/S1679-45082015RW3102)
Supplement: Translation

# Translation: Impacto dos efeitos tardios da radiação em crianças sobreviventes de câncer: revisão integrativa

**Authors:** Cibeli Fernandes Coura, Patrícia Cláudia Modesto

**Affiliations:** 1 Hospital Israelita Albert Einstein, São Paulo, SP, Brasil.

**Keywords:** Neoplasias/radioterapia, Neoplasias/efeitos da radiação, Criança

## Abstract

Objetivamos identificar os efeitos tardios da exposição à radiação em crianças sobreviventes do câncer. Foi realizada uma revisão integrativa de literatura, nas bases de dados MEDLINE e LILACS, e no suporte eletrônico SciELO. Foram incluídos artigos nos idiomas português e inglês, publicados nos últimos 10 anos, utilizando os descritores “neoplasias/*neoplasms” AND “*radioterapia*/radiotherapy” AND “*radiação*/radiation”*. Após análise, 14 artigos, publicados em nove revistas conceituadas, atenderam os critérios de inclusão. As publicações foram divididas em duas categorias: “Efeitos tardios endócrinos” e “Efeitos tardios não endócrinos”. Com o aumento nas taxas de sobrevida em crianças sobreviventes do câncer, foi observado o impacto dos efeitos tardios referentes à exposição da radiação, e decorrentes do tratamento e de exames radiodiagnósticos. Sobreviventes de câncer na infância estiveram expostos a diversos efeitos tardios e devem ter um seguimento precoce e regular, ainda que expostos a doses baixas de radiação.

## INTRODUÇÃO

A incidência de câncer no mundo tem aumentado consideravelmente, tornando-se um problema mundial de saúde pública. Segundo a Organização Mundial da Saúde, estima-se que até o ano de 2030 sejam diagnosticados 21,4 milhões de novos casos de câncer. No Brasil, as perspectivas para o ano 2014 e 2015 apontavam uma ocorrência de aproximadamente 576 mil novos casos de câncer.^1^

Os tumores pediátricos representam de 1 a 3% do total de casos de câncer no mundo. No Brasil, estimou-se que, em 2014 e 2015, seriam identificados 11.840 novos casos de câncer, representando uma das principais causas de morte entre crianças e adolescentes.^1^

O tratamento contra o câncer pode ser feito por meio de cirurgia, quimioterapia e radioterapia (RT). Com os avanços tecnológicos nas modalidades terapêuticas, observou-se importante impacto na taxa de sobrevida global desses pacientes. Atualmente, 80% deles podem ser curados, se diagnosticados precocemente.^2^

A RT é uma das modalidades terapêuticas mais utilizadas para diminuir ou impedir o crescimento tumoral. A exposição em tecidos normais, entretanto, pode resultar em câncer. A radiação ionizante lesa os tecidos humanos, produzindo modificações químicas e biológicas irreversíveis, e resultando em morte celular.^3^

Concomitante a essa modalidade de tratamento, tem-se observado o impacto dos efeitos tardios a curto e longo prazos, referentes à exposição da radiação, e decorrentes do tratamento e de exames radiodiagnóstico em crianças e adolescentes. Os efeitos colaterais dessa exposição podem causar alterações endócrinas e não endócrinas, modificando o estado nutricional dos indivíduos, que ficam mais suscetíveis a complicações.^4^

Dessa maneira, é necessário ter um seguimento precoce e regular, que possibilite o diagnóstico desses efeitos colaterais tardios, proporcionando, assim, uma melhora na qualidade de vida, dada a alta taxa de sobrevida desses pacientes.

## OBJETIVO

Identificar os efeitos tardios da exposição à radiação em crianças sobreviventes do câncer.

## MÉTODOS

Trata-se de uma pesquisa realizada por meio de revisão integrativa, que incluiu literatura teórica e empírica, bem como estudos com diferentes metodologias. Para a elaboração desta pesquisa, foram percorridas as seguintes etapas: estabelecimento da hipótese e objetivo da revisão integrativa; determinação dos critérios de inclusão e exclusão para a seleção da amostra; definição das informações a serem extraídas dos artigos; análise dos resultados; discussão e interpretação dos resultados; e apresentação da revisão.^5^

A questão norteadora escolhida foi: quais são os efeitos tardios da exposição à radiação em crianças sobreviventes do câncer?

Foram realizadas pesquisas em periódicos eletrônicos nas bases de dados *Medical Literature Online* (MEDLINE) e Literatura Latino-Americana e do Caribe de Informação em Ciências da Saúde (LILACS), e no suporte eletrônico *Scientific Eletronic Library Online* (SciELO).

Para a busca dos artigos, foram utilizados os descritores: “neoplasias/*neoplasms” AND “*radioterapia*/radiotherapy” AND “*radiação*/radiation”.* Os descritores encontram-se nos Descritores em Ciências da Saúde da BIREME.

Os critérios de inclusão para seleção da amostra foram: artigos disponível *on-line* com texto completo, nos idiomas português e inglês, publicados num período de 10 anos (2003 a 2013) e que abrangessem os efeitos tardios da exposição à radiação em crianças sobreviventes do câncer. Foram excluídos os estudos que estavam em duplicidade nas bases de dados e que não atendessem ao objetivo da pesquisa.

Ao cruzar os descritores nas bases de dados *on-line*, foram encontrados 452 artigos, que inicialmente foram avaliados pelo título e pelo resumo; quando apresentaram compatibilidade com a pergunta norteadora e com os critérios de inclusão, foram lidos na íntegra. Após análise crítica, a amostra final foi constituída por 14 artigos.

Para a coleta de dados, foi elaborado pelos autores desta pesquisa um roteiro estruturado, composto pelos seguintes dados: identificação dos artigos, tipo de publicação, idioma, autor, ano de publicação, título, população, objetivo, resultados e conclusões.

Os dados foram analisados segundo seus conteúdos e, após análise do material, os artigos foram agrupados e identificados em categorias temáticas.

A apresentação dos resultados e a discussão dos dados foram feitas de maneira descritiva e em tabelas, possibilitando ao leitor a aplicabilidade dos resultados da revisão integrativa elaborada.

## RESULTADOS

Dos 452 artigos inicialmente avaliadas pelo título e resumo, 61 foram lidos na íntegra por apresentarem compatibilidade com a pergunta norteadora e com o objetivo da pesquisa. Após análise crítica, foram excluídos 44 artigos por não atenderem aos critérios de inclusão e três por dupla indexação. Assim, fizeram parte desta revisão 14 artigos.

Dentre os artigos incluídos, 12 foram encontrados na base de dados MEDLINE (86%), um no LILACS (7%) e um na SciELO (7%). Em relação ao ano de publicação, dois foram em 2012, três em 2011, quatro em 2010, dois em 2009, um em 2008, um em 2006, e um em 2005.

Houve predominância do idioma inglês nas publicações, com 12 artigos (86%).

Ao analisar as características do tipo de estudo, seis eram revisões retrospectivas (duas de abordagem longitudinal e quatro de coorte, sendo uma abordagem observacional e três longitudinais), dois artigos eram revisões de literatura, e dois revisões sistemáticas.

Os artigos selecionados foram publicados em sete revistas conceituadas: *Cancer* (três artigos); Arquivo Brasileiro de Endocrinologia & Metabologia (dois artigos), *Radiology eRadiation Research* (dois artigos); *Archives of Internal Medicine* (um artigo); *Pediatr Blood Cancer* (dois artigos) e *The Lancet* (um artigo).

A tabela 1 mostra as categorias e o número de publicações identificadas neste estudo.

**Tabela 1:** Descrição das categorias

Categoria	Número de publicações
Efeitos tardios endócrinos	7
Efeitos tardios não endócrinos	10
Total	17

As publicações foram divididas em duas categorias temáticas: “Efeitos tardios endócrinos” e “Efeitos tardios não endócrinos”. Dos 14 artigos selecionados, 7 fizeram parte da categoria “Efeitos tardios endócrinos” e 10 da “Efeitos tardios não endócrinos” (3 artigos apareceram nas duas categorias).

No quadro 1 estão apresentados os artigos de acordo com autores, ano de publicação, título, tipo de publicação e resultado.

**Quadro 1:** Descrição dos artigos

Autor	Título	Tipo de estudo	Resultados
Bonato et al.^(3)^	Alterações tireoidianas associadas à radiação externa em crianças e adolescente	Revisão de literatura	A exposição de crianças à radiação, oriunda de exames radiodiagnósticos, causou hipotireoidismo e câncer de tireoide
Couto-Silva et al.^(4)^	Sequelas endócrinas da RT no tratamento do câncer na infância e adolescência	Revisão de literatura	Altas doses de radiação causaram deficiência de hormônio do crescimento, obesidade, hipotireoidismo, disfunção gonadal e perda acentuada da estatura
Armstrong et al.^(6)^	*Long-term effects of radiation exposure among adult survivors of childhood cancer: results from the childhood cancer survivor study*	Revisão retrospectiva longitudinal	A RT esteve associada com o aumento do risco de mortalidade tardia, neoplasias, obesidade, disfunção pulmonar, insuficiência cardíaca e hipotireoidismo
Cheuk et al.^(7)^	*Prognostic factors and long-term outcomes of childhood nasopharyngeal carcinoma*	Revisão retrospectiva	Pacientes com carcinoma nasofaríngeo que receberam RT >30 Gy para o tumor primário desenvolveram neoplasia subsequente e morbidades
Bhatti et al.^(8)^	*Risk of second primary thyroid cancer after radiotherapy for a childhood cancer in a large cohortstudy: an update from the childhood cancer survivor study*	Coorte retrospectivo longitudinal	O risco de um segundo câncer primário de tireoide em sobreviventes de câncer na infância aumentou com dose de radiação até 20 Gy, com risco relativo com pico 14 vezes maior
Meacham et al.^(11)^	*Diabetes mellitus in long-term survivors of childhood cancer. Increased risk associated with radiation therapy: a report for the childhood cancer survivor study*	Coorte retrospectiva longitudinal	Foram comparados três tipos de RT (ICT, abdominal e craniana) associados ao *diabetes mellitus*. Os tratados ICT foram sete vezes mais propensos ao *diabetes mellitus*
Wallace^(12)^	*Oncofertility and preservation of reproductive capacity in children and young adults*	Revisão retrospectiva	Crianças expostas a ICT, ou abdominal, ou pélvica tiveram a fertilidade prejudicada em ambos os sexos
Grewal et al.^(13)^	*Auditory late effects of childhood cancer therapy: a report from the children’s Oncology Group*	Revisão sistemática	A RT craniana em altas doses resultou em ototoxicidade, prejudicando a qualidade de vida em crianças
Motosue et al.^(15)^	*Pulmonary function after whole lung irradiation in pediatric patients with solid malignancies*	Revisão retrospectiva longitudinal	A irradiação no pulmão trouxe complicações pulmonares, e quem recebeu impulsos de radiação teve aumento de morbidade e metástase
Jones et al.^(17)^	*Renal late effects in patients treated for cancer in childhood: a report from the Children’s Oncology Group*	Revisão sistemática	Indivíduos tratados de câncer na infância com radiação tiveram risco de insuficiência renal. Doses >20 Gy resultaram em nefropatia
Ritchey et al.^(18)^	*Late effects on the urinary bladder in patients treated for cancer in childhood: a report from the Children’s Oncology Group*	Revisão de literatura retrospectiva	Altas doses da radiação na região pélvica aumentaram o risco de anormalidades na função da bexiga como cistite hemorrágica, fibrose e bexiga neurogênica
Taylor et al.^(19)^	*Population-based risks of CNS tumors in survivors of childhood cancer: the British Childhood Cancer Survivor Study*	Coorte retrospectiva longitudinal	O aumento do risco de um segundo tumor primário no sistema nervoso central foi demonstrado após exposição à radiação nos tecidos da meninge
Kleinerman^(20)^	*Cancer risks following diagnostic and therapeutic radiation exposure in children*	Revisão retrospectiva	A exposição a múltiplos exames de imagens aumentou o risco de câncer em crianças, associada à radiação vinda desse procedimento
Pearce et al.^(21)^	*Radiation exposure from CT scans in childhood and subsequent risk of leukaemia and brain tumours: a retrospective cohort study*	Coorte retrospectivo observacional	O uso de TCs em crianças acumulou doses de radiação de 50 Gy, podendo triplicar o risco de leucemia, e doses de 60 Gy pode triplicar o risco de câncer no cérebro

RT: radioterapia; ICT: irradiação corporal total; TC: tomografia computadorizada; CNS: *central nervous system.*

## DISCUSSÃO

### Efeitos tardios endócrinos

As sequelas endócrinas foram descritas após a exposição à irradiação em pacientes submetidos à terapêutica oncológica e se manifestam como hipotireoidismo, deficiência de hormônio de crescimento (GH), obesidade, diabetes e distúrbio gonadal.^3,4,6-12^

As tireoidopatias são as complicações endócrinas mais frequentes. Em população de crianças irradiadas na região, o hipotireoidismo foi encontrado em 47% dos pacientes, 27 anos após tratamento, sendo que metade desses casos foi manifestada até 5 anos após a RT.^3,4^ O hipotireoidismo também foi identificado em 34% dos 1.791 sobreviventes de linfoma Hodgkin com risco relativo (RR) de 17,1.^6^ Outros autores mostraram que 30% dos pacientes que receberam doses de 35 a 45 Gy de RT, e 50% dos que receberam doses >45 Gy deveriam ter hipotireoidismo 20 anos após o tratamento, e o risco de nódulos estimado foi 27 vezes maior.^4,6^ A incidência cumulativa, numa estimativa de 15 anos de hipotireoidismo primário, para quem recebeu dose RT >60 Gy foi 77,2%; com dose de 50 a 60 Gy, foi de 47,4%; e com dose <50 Gy, foi de 9,5%.^7^ Em curto prazo, foi observado em 39% das crianças tratadas com radiação em diferentes áreas corporais, nas quais o hipotireoidismo surgiu, em média, 3,5±1,9 anos após a RT.^3^

Nódulos adenomatosos foram observados em 30 a 90% na irradiação de cabeça e pescoço, e o carcinoma papilar foi o mais comum em 78% dos casos de segundo câncer de tireoide.^3^ Numa coorte com 17.980 sobreviventes de câncer na infância, o RR foi de 4,6, com intervalo de confiança de 95% (IC 95%) para o aparecimento de segundo câncer de tireoide, quando comparados aos sobreviventes não tratados com RT.^3^ Em 119 crianças tratadas para um câncer na infância, todas tiveram um segundo câncer primário de tireoide e o risco aumentou com a dose de radiação entre 20 e 25 Gy, com RR com pico de 14,6 vezes maior com IC 95% de 6,8-31,5.^8^

A RT craniana também levou à deficiência do GH. Dentre os pacientes que receberam doses de 24 Gy, 56% tiveram deficiência de GH e, entre os que receberam doses >45 Gy, 100% tiveram deficiência.^4^ A diminuição de GH foi observada logo após 18 meses nos que receberam doses de 31 a 42 Gy, e 60% tiveram deficiência de GH após 2 anos. Uma vez estabelecida a deficiência de GH, ocorreu a desaceleração acentuada da estatura em doses >45 Gy; já nas doses <24 Gy, a desaceleração foi inconstante. A perda da estatura com encurtamento do tronco foi constatada em pacientes que receberam irradiação espinhal com dose >20 Gy.^4^ O mesmo foi confirmado em outro estudo, no qual 100% das crianças que receberam doses >30 Gy tiveram deficiência de GH e a deficiência isolada de GH pode ocorrer com baixa dose de 18 Gy. Além disso, as crianças tiveram maior risco de baixa estatura, confirmando, assim, a desaceleração do crescimento nesses pacientes.^9^

A obesidade foi um preditor de risco para diabetes, dislipidemia, hipertensão e doença cardiovascular, e esteve associada com a radiação craniana em doses de 20 a 24 Gy.^6^ Pacientes que receberam RT craniana com dose de 20 Gy (média 24 Gy) tiveram risco aumentado de sobrepeso e obesidade, e, naqueles que receberam dose >45 Gy, a composição corporal aumentou, confirmando que o ganho ponderal era dose-dependente.^10^

Sobreviventes que receberam irradiação abdominal tiveram 2,7 vezes mais chance de desenvolver *diabetes mellitus* comparados aos não tratados, e aqueles que receberam irradiação corporal total (ICT) foram 7,2 vezes mais propensos a ter *diabetes mellitus*.^11^

A disfunção gonadal após a RT craniana, abdominal e pélvica ou ICT prejudicou a fertilidade em ambos os sexos.^4,12^ A puberdade precoce apareceu após doses 18 a 24 Gy. Doses <4 Gy não levaram à disfunção ovariana permanente; doses >20 Gy e ICT levaram à falência ovariana na maioria das crianças e adolescentes.^4^ Dentre as pacientes que, durante a infância, receberam doses de 10 a 15,75 Gy, após tomografia cranioencefálica, 90% tiveram insuficiência ovariana; naquelas que receberam irradiação abdominal de 20 a 30 Gy, 97% tiveram a insuficiência, aumentado o risco de menopausa precoce. A insuficiência ovariana e a menopausa precoce foram observadas na coorte do *ChildhoodCancer Survivor Study* (CCSS) com 14 mil sobreviventes de câncer na infância tratadas com irradiação pélvica. Essa exposição aumenta o risco durante a gravidez de aborto espontâneo, parto prematuro e baixo peso ao nascer.^12^

Nos homens, doses baixas de radiação, entre 0,1 e 1,2 Gy, podem prejudicar a espermatogênese; e doses de >4 Gy causaram azoospermia permanente. As células de Leydig foram mais resistente que as células germinativas, e seu dano só foi observado em doses >20 Gy em menino pré-púberes e em doses de 30 Gy em meninos pós-púberes.^12^

### Efeitos tardios não endócrinos

A RT teve papel essencial no tratamento de várias neoplasias infantis, aumentando a sobrevida desses pacientes, porém também tem sido associada a vários efeitos deletérios, como perda auditiva e disfunção de vários órgãos (como pulmão, coração, rim e bexiga), assim como o desenvolvimento de segunda neoplasia.^6,7,13-19^

Verificou-se que a RT craniana com doses >32 Gy causou ototoxicidade em pacientes pediátricos, com perda gradual da audição, manifestando-se em meses ou anos após a exposição. A ototoxicidade também pode ser vista 34% dos pacientes, e os graus 3 e 4 foram relatados em 9% dos que receberam RT antes da quimioterapia.^13^ O CCSS confirmou a perda auditiva após doses >30 Gy (RR: 7,5; IC 95%: 3,6-15,5). Com doses >50 Gy, o risco aumentou (RR: 10,7; IC 95%: 5,2-22,2), afirmando, assim, a ototoxicidade.^14^

A radiação no tórax e ICT causaram toxicidade pulmonar com RR de 4,3 para a fibrose pulmonar, RR de 2,2 para pneumonia recorrente e RR de 2,0 para tosse crônica.^6^ Em 48 sobreviventes de tumores sólidos pediátricos com acompanhamento médio de 9,7 anos tratados com irradiação do pulmão inteiro, com dose média de 12 Gy, houve uma diminuição significativa na capacidade pulmonar. E, ainda, 17 destes pacientes receberam radiação por impulso pulmonar focal, com dose média de 18 Gy, aumentando a morbidade pulmonar, além de doença metastática.^15^

A exposição da radiação no coração com doses 15 Gy aumentou o risco em duas a seis vezes de insuficiência cardíaca congestiva e infarto do miocárdio, se comparada aos sobreviventes não expostos. Quem recebeu radiação no tórax, na coluna vertebral ou ICT teve risco três vezes maior de morte cardíaca (RR: 3,3; IC95%: 2,0-5,5).^6^ Também foi possível confirmar que, na RT no coração com dose 20 Gy, o risco de doença cardíaca foi 4,40 vezes maior (IC95%: 1,0-17,5) comparada aos não expostos, e o RR de insuficiência cardíaca de quem recebeu dose média de 5 a 20 Gy foi de RR de 2,52, com IC95% de 0,96-6,6. Já nas dose >20 Gy, o RR foi 5,65 e IC 95% de 1,45-22,0, confirmando o alto risco de morbimortalidade.^16^

A função renal foi avaliada em pacientes tratados de câncer na infância e verificou-se que pacientes suscetíveis a radiação no rim, nefropatia ou nefrite surgiram após um período de 3 a 12 meses, manifestando-se por meio da hipertensão, proteinúria, anemia e insuficiência renal. Doses <18 Gy no rim raramente causaram lesões graves; já doses >20 Gy resultaram em nefropatia significativa.^17^

A cistite hemorrágica induzida pela radiação pode ser aguda ou crônica, com doses >30 Gy na bexiga inteira e doses >60 Gy em uma porção. De 11 crianças tratadas para rabdomiossarcoma pélvico após 6 anos, 7 foram irradiadas apresentando enurese e capacidade anormal da bexiga. Em outros 40 pacientes tratados com a mesma doença, 9, dos 19 irradiados, apresentaram problemas na bexiga, quem recebeu dose 40 Gy teve maior incidência de sintomas urinários e todos que receberam doses >50 Gy tiveram complicações na bexiga. A RT >50 Gy na coluna lombar ou no sacro trouxeram como consequências danos nos nervos periféricos que resultaram em bexiga neurogênica.^18^

A neoplasia subsequente foi observada em 5 dos 59 pacientes com carcinoma de nasofaringe que receberam RT com doses de 30 a 70 Gy.^7^ Na coorte CCSS, 298 sobreviventes desenvolveram neoplasias malignas subsequentes à exposição da radiação com a incidência cumulativa de 3,2% em 20 anos após o diagnóstico primário de câncer infantil. Estudo com 6.068 mulheres sobreviventes constatou que 95 delas tiveram câncer de mama secundário após a radiação no tórax com taxa de incidência padronizada (TIP) de 24,7 e IC 95%: 19,3-31,0; comparadas com as que não receberam RT, a TIP foi 4,8 (IC95%:2,9-7,4) e, ainda, observaram-se que doses de 40 Gy no tórax tiveram risco de câncer de mama aumentado em 11 vezes.^6^ A literatura também identificou 247 segundas neoplasias primárias do sistema nervoso central, sendo 137 meningeomas, 73 gliomas e 37 outros tipos de cânceres. A TIP foi 14,3 (IC95%: 10,9-18,7) após RT; comparada aos que não receberam RT, a TIP foi 6,1 (IC 95%: 3,1-11,0). Em pacientes irradiados com dose 40 Gy, RR ajustado de meningeoma foi de 479 vezes maior do que os não expostos.^19^

### Efeitos tardios de exames diagnósticos

Exames diagnósticos e sua associação com câncer já foram relatados.^3,20,21^ Embora as doses de radiação de um único procedimento possam ser baixas, os pacientes pediátricos fazem múltiplos exames, resultando em doses cumulativas relativamente altas. Em relação à exposição a radiação no tórax por múltiplas fluoroscopias usadas para monitorar tratamento de tuberculose em meninas adolescentes e mulheres jovens, com dose média da exposição fracionada de 0,79-2,1 Gy, o aparecimento de câncer de mama surgiu 15 anos após a exposição e até 50 anos mais tarde ele permaneceu elevado.^20^ Em um exame de dosimetria realizado em crianças com tomografia na região cervical, a tireoide pode receber doses de 15,2 a 52 Gy, o que poderia aumentar os casos de malignidade em mais de 390 a cada milhão de pessoas expostas.^3^ Em uma coorte com crianças e adultos jovens sem diagnóstico de câncer, que utilizaram TC, 74 dos 178.604 pacientes foram diagnosticados com leucemia e 135 dos 176.587 pacientes foram diagnosticados com tumor cerebral. O RR de leucemia em pacientes que receberam dose média de 51,13 mGy foi de 3,18, comparado a quem recebeu dose <5 mGy. Já o RR de tumor no cérebro para pacientes que receberam dose média 60,42 mGy foi 2,82, e quem recebeu dose média de 104,16 mGy teve RR de 3,32 em comparação aos que receberam dose <5 mGy. TC em crianças <15 anos com doses cumulativas de radiação de 50 mGy podem triplicar o risco de leucemia, e doses cumulativa de 60 mGy podem triplicar o risco de tumor cerebral.^21^

O estudo realizado apresentou limitação na metodologia de busca quanto à escolha dos descritores e ao período, gerando uma busca muito ampla e pouco sensível. Outro parâmetro esteve relacionado com a dificuldade de obter mais amostras, devido à indisponibilidade do acesso aos artigos não gratuitos. Todos esses fatores contribuíram para as limitações do estudo e, certamente, uma amostra mais significativa e representativa permitiria uma maior validade.

## CONCLUSÃO

Os autores mostram que, apesar dos avanços terapêuticos contra o câncer, têm-se observado os efeitos colaterais alarmantes apresentados a curto e longo prazos referentes à exposição da radiação, advindos do tratamento e de exames diagnósticos. Observou-se que os sobreviventes estiveram expostos a diversos efeitos tardios, como hipotireoidismo, deficiência de GH, obesidade, *diabetes mellitus*, infertilidade, perda auditiva, disfunção de diversos órgãos, como pulmão, coração, rim e bexiga, além do desenvolvimento de segunda neoplasia. Assim, devemos enfatizar a importância de um seguimento precoce e regular aos pacientes expostos à radiação na infância mesmo em baixas doses, a fim de possibilitar o diagnóstico e o tratamento desses efeitos colaterais, resultando em uma melhor qualidade de vida.
